# The key role of contact time in elucidating the mechanisms of enhanced decontamination by Fe^0^/MnO_2_/sand systems

**DOI:** 10.1038/s41598-021-91475-x

**Published:** 2021-06-08

**Authors:** Viet Cao, Ghinwa Alyoussef, Nadège Gatcha-Bandjun, Willis Gwenzi, Chicgoua Noubactep

**Affiliations:** 1 Faculty of Natural Sciences, Hung Vuong University, Nguyen Tat Thanh Street, Viet Tri, Phu Tho 35120 Vietnam; 2 Angewandte Geologie, Universität Göttingen, Goldschmidtstraße 3, 37077 Göttingen, Germany; 3 Department of Chemistry, Faculty of Science, University of Maroua, BP 46, Maroua, Cameroon; 4 Biosystems and Environmental Engineering Research Group, Department of Agricultural and Biosystems Engineering, University of Zimbabwe, P.O. Box MP167, Mt. Pleasant, Harare Zimbabwe; 5 Centre for Modern Indian Studies (CeMIS), Universität Göttingen, Waldweg 26, 37073 Göttingen, Germany; 6 Department of Water and Environmental Science and Engineering, Nelson Mandela African Institution of Science and Technology, P.O. Box 447, Arusha, Tanzania

**Keywords:** Environmental sciences, Materials science

## Abstract

Metallic iron (Fe^0^) has shown outstanding performances for water decontamination and its efficiency has been improved by the presence of sand (Fe^0^/sand) and manganese oxide (Fe^0^/MnO_x_). In this study, a ternary Fe^0^/MnO_x_/sand system is characterized for its discoloration efficiency of methylene blue (MB) in quiescent batch studies for 7, 18, 25 and 47 days. The objective was to understand the fundamental mechanisms of water treatment in Fe^0^/H_2_O systems using MB as an operational tracer of reactivity. The premise was that, in the short term, both MnO_2_ and sand delay MB discoloration by avoiding the availability of free iron corrosion products (FeCPs). Results clearly demonstrate no monotonous increase in MB discoloration with increasing contact time. As a rule, the extent of MB discoloration is influenced by the diffusive transport of MB from the solution to the aggregates at the bottom of the vessels (test-tubes). The presence of MnO_x_ and sand enabled the long-term generation of iron hydroxides for MB discoloration by adsorption and co-precipitation. Results clearly reveal the complexity of the Fe^0^/MnO_x_/sand system, while establishing that both MnO_x_ and sand improve the efficiency of Fe^0^/H_2_O systems in the long-term. This study establishes the mechanisms of the promotion of water decontamination by amending Fe^0^-based systems with reactive MnO_x_.

## Introduction

Since the 1950s the world is conventionally divided into two groups with regards to the access to safe drinking water: (i) developed countries striving for selecting the best available technology for water treatment, and (ii) developing countries striving for making some appropriate technologies available for their mostly low-income and rural populations^1–3^. In the meantime, cities have grown, but drinking water systems in peri-urban areas are comparable to that of rural environments without piped water^4–6^. Appropriate technologies are essentially considered as interim solutions wherever a centralized water supply is not yet available^1^. Factors relevant for selecting an appropriate solution for safe drinking water supply include^1,7,8^: (i) Simplicity in operation (no special skilled personnel), (ii) robustness (no frequent break downs), (iii) affordability (low installation and operation costs), (iv) ability to function without electricity, and (v) use of local skills and readily available resources. Research during the past two decades has rediscovered filtration systems based on metallic iron (Fe^0^) as an affordable, applicable, and efficient water treatment technology for decentralized water supply (e.g. households and small communities)^3,7,9–13^. Such Fe^0^ filters are only sustainable upon admixing Fe^0^ with other aggregates like granular activated carbon, biochar, gravel, magnetite (Fe_3_O_4_), manganese oxides (MnO_x_), pyrite (FeS_2_), and sand^10,14,15^.

There are two fundamental challenges in designing Fe^0^ filtration systems: (i) “reactivity loss” and (ii) permeability loss^16–20^. Permeability loss is not addressed herein. For the presentation, it suffices to recall that this issue has been partly resolved in earlier studies demonstrating that only hybrid Fe^0^ filters are sustainable^14,21^. The remaining task is a temporal issue regarding the reaction kinetics of Fe^0^ corrosion, which is material-specific and has received limited attention^22–24^. Reactivity loss is the expression of the inherent time-dependent decrease of the Fe^0^ corrosion rate as well-documented in the corrosion literature and referred to as ‘passivation’^25–28^. However, “reactivity loss” has been introduced in the post-1990 literature to characterize the limited electron transfer from the metal body to some dissolved contaminants^16,19^. Given that under natural conditions Fe^0^ is corroded only by protons from water dissociation (Eq. 1)^29^, Miyajima and Noubactep^30^ argued that reactivity loss is a mirage. In fact, “reactivity loss” has also occurred in Fe^0^-based permeable reactive barriers successfully working for up to two decades^31–34^. On the other hand, Roh et al.^35^ reported on Fe^0^ specimens from World War I still corroding in soils. Clearly, it can be argued that the old motto “rust never rests” is valid for Fe^0^ filters where corrosion additionally occurs under immersed conditions. The question is, how to ensure that Fe^0^ oxidation with changing corrosion rates still secures clean water in the long-term?

1 $${\text{Fe}}^{0} + {\text{ 2 H}}^{ + } \Rightarrow {\text{Fe}}^{{{2} + }} + {\text{ H}}_{{2}}$$

2 $${\text{Fe}}^{0} + {\text{ MnO}}_{{2}} + {\text{ 4 H}}^{ + } \Rightarrow {\text{Mn}}^{{{2} + }} + {\text{ Fe}}^{{{2} + }} + {\text{ 2 H}}_{{2}} {\text{O}}$$

3a $${\text{2 Fe}}^{{{2} + }} + {\text{ MnO}}_{{2}} + {\text{ 4 H}}^{ + } \Rightarrow {\text{Mn}}^{{{2} + }} + {\text{ 2 Fe}}^{{{3} + }} + {\text{ 2 H}}_{{2}} {\text{O}}$$

3b $${\text{2 Fe}}^{{{2} + }} + {\text{ MnO}}_{{2}} + {\text{ 2 H}}_{{2}} {\text{O}} \Rightarrow {\text{Mn}}^{{{2} + }} + {\text{ 2 FeOOH }} + {\text{ 2 H}}^{ + }$$

**Figure 1:**
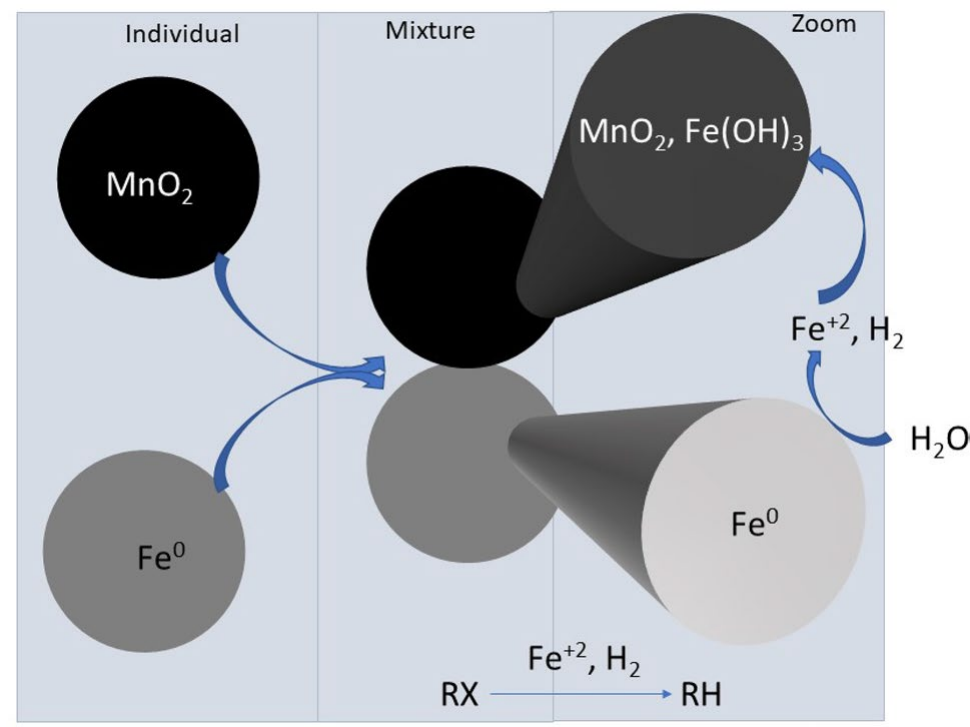
Scheme showing the pathways of contaminant reductive transformations in Fe^0^/MnO_2_/H_2_O systems. Only water has access to the metal surface. Fe^2+^ and H_2_ are stand-alone reducing agents. MnO_2_ and other relevant dissolved species (e.g. RX) are reduced by Fe^2+^ and H_2_. Upon the oxidation of Fe^2+^, various solid iron hydroxides/oxides (e.g. Fe(OH)_3_) precipitate and act as contaminant scavengers. RX stands for an halogenated hydrocarbon. The key information is that MnO_2_ is not reduced by Fe^0^.

During the past decade, substantial experiences have been accumulated on increasing the efficiency of Fe^0^/H_2_O systems by admixing Fe^0^ with other materials (Table 1)^15,36^. However, these efforts were mostly misled by the misconception that Fe^0^ is a reducing agent^36^. Fortunately, available data can be re-interpreted based on the chemistry of the system. It suffices to consider that reduction is not a relevant contaminant removal mechanism, and that contaminant reduction is never mediated by electrons from the metal body^11^. For example, MnO_2_ is not reduced by Fe^0^ (Eq. 2), but rather by Fe^2+^ (Eq. 3a) (Fig. 1). Equation 3b depicts that MnO_2_ reductive dissolution by Fe^2+^ induces acidification of the system (releases protons). O_2_ and other dissolved species are equally reduced by Fe^2+^ and other reductive species present in the Fe^0^/H_2_O system (e.g., H_2_, Fe_3_O_4_, green rust)^37–40^. Thus, it is established that contaminants are reduced by an indirect mechanism (Fig. 1), and that this process continues even after virtual surface passivation (which is thus not a “loss of their reactivity”). Successful efforts to overcome Fe^0^ passivation include the addition of gravel^41,42^, magnetite^15,43^, MnO_x_^44,45^, pyrite^36,46^, and sand^47,48^.

**Table 1:** Summary of the operating mode of some representative aggregates relevant for hybrid Fe^0^ systems for water treatment. Their status according to the state-of-the-art knowledge on the Fe^0^/H_2_O system is given as comments. Questioned aspects have been documented in short-term laboratory experiments, but are not likely to be valid when the aggregates are coated with iron corrosion products (FeCPs).

Aggregate	Assigned function	Comments
Fe^0^	Generates contaminant scavengers (FeCPs)	Confirmed
Fe^0^	Donates electrons to contaminants	Disproved
GAC	Sustains iron corrosion (Fe^0^/GAC cells)	Questioned
GAC	Accumulates contaminants for reduction by Fe^0^	Questioned
GAC	Sustains long-term iron corrosion	Confirmed
Sand	Scavengers of FeCPs (in-situ coating)	Confirmed
Sand	Impairs the efficiency of the Fe^0^ system	Disproved
Sand	Sustains long-term iron corrosion	Confirmed
MnOx	Corrodes Fe^0^ (as cathodic reaction)	Disproved
MnOx	Scavengers of Fe^2+^	Confirmed
MnOx	Sustains long-term iron corrosion	Confirmed

The presence of inert sand improves the efficiency of even batch Fe^0^/H_2_O systems for water treatment^49^. However, the studies testing other reactive materials have not considered inert systems (e.g., sand) as operational references. Only Ndé-Tchoupé et al.^50^ did such a comparison. However, the objective was to test pozzolan as an alternative filling material to sand for Fe^0^ filters. In other words, while testing magnetite (Fe_3_O_4_) as admixing agent for the reductive transformation of contaminants^43^, a reference Fe^0^/sand should have been considered in parallel experiments. The inclusion of an operational reference enables a better understanding of the specific action of the reactive additive (here Fe_3_O_4_). Following the science of aqueous iron corrosion under environmental conditions^25,28^, this study premises that iron passivation is delayed by avoiding the precipitation of iron corrosion products in the vicinity of the metal. Thus, Fe^2+^ and Fe^3+^ ions are consumed instead of coating sand in Fe^0^/sand systems, and Fe^2+^ ions are additionally consumed in the reductive dissolution of MnO_x_ (Eq. 2) in the Fe^0^/MnO_x_/sand systems (Fig. 1). Note that all other aggregates including Fe_3_O_4_ and granular activated carbon are in-situ coated by FeCPs like sand and the postulated effects are not realizable in the long-term (Table 1).

Fe^0^ is used to efficiently remove various contaminants such as turbidity, pathogens, and dissolved species from aqueous solutions^51–60^. Chemical pollutants in the aqueous phase can be ions, molecules, and colloids. For reducible dissolved species, there is a trend to consider Fe^0^-based materials (E^0^ = − 0.44 V) as (strong) reducing agent^51,56,60^, and contaminant reductive transformation as an electrochemical process^58^. It is evident that colloids, pathogens, and suspended solids (turbidity) are not removed by any chemical reaction. Rather, they are removed via coagulation and co-precipitation. The previous text has already demonstrated that no electrochemical contaminant reduction is possible in a Fe^0^/H_2_O system^58^. Yet, published results using the Fe^0^/MnO_2_ mixtures are mainly premised on the wrong assumption that contaminant removal occurs via reduction by electrons from Fe^0^ (Fig. 1) (Table 1)^6,54,57^. Accordingly, there is still a need to further clarify the role of MnO_2_ in sustaining the efficiency of Fe^0^/H_2_O systems for water treatment^59^. In particular, there is need to elucidate how insoluble Fe(OH)_3_ contribute to the co-precipitation of pollutants from the aqueous phase.

The objective of this study is to investigate the impact of MnO_x_ addition on the efficiency of Fe^0^/H_2_O systems for MB discoloration as a function of the experimental duration (contact time). The specific objective is to confirm the suitability of ‘MB discoloration’ as powerful tool for the characterization of decontaminantion processes in Fe^0^/H_2_O systems while using MnO_x_ and sand to control the availability of ‘free’ FeCPs. The extent of MB discoloration is investigated in five different systems: (i) Fe^0^ alone, (ii) sand alone, (iii) Fe^0^/sand, (iv) Fe^0^/MnO_x_, and (v) Fe^0^/MnO_x_/sand for 7, 18, 25 and 47 days. A comparison of the results from the five systems provides critical information on the contaminant removal mechanisms and the role of MnO_x_.

##  Materials and methods

### The theory of iron and manganese cycle in a Fe^0^/MnO_x_/sand system

Initially (t_0_ = 0), when Fe^0^, MnO_x_ and sand are put into the solution, there is no dissolved iron and no dissolved manganese in the system (Table 2). At t > t_0_, Fe^0^ is dissolved by protons (water) to generate H_2_ and Fe^2+^ (Eq. 1). Fe^2+^ induces the reductive dissolution of MnO_x_ (Eq. 3)^61–65^. At t > t_0_, the Fe^0^/MnO_x_/sand system hosts dynamic processes which might continue after Fe^0^ depletion. In fact, the mixture of Fe and MnO_x_ minerals is a very complex reactive system that has been investigated for more that a century^66–68^. The uniqueness of the Fe^0^/MnO_x_/sand system is that Fe minerals are generated in-situ and are comparatively more reactive than aged minerals like goethite or hematite. Because the pH of the system is larger than 5.0, in the absence of ligands, Fe and Mn hydroxides have very low solubility and precipitate not far away from their points of nucleation^65,69^. The dynamics within the Fe^0^/MnO_x_/sand entail a series of interchanges of iron and manganese from older to younger forms as follows: (i) dissolution of Fe^0^ and MnO_x_, (ii) migration of Fe^2+^, Fe^3+^ and Mn^2+^ from the areas of their generation to areas where precipitation will occur, and (iii) precipitation in one or more forms of iron and manganese hydroxide.

**Table 2:** Time-dependent inventory of reactive species in the four investigated systems. t_0_ corresponds to the start of the experiment, while t_∞_ corresponds to the time required for Fe^0^ depletion. It is assumed that MnO_2_ is quantitatively converted to MnOOH without impact on MB discoloration. FeCPs: Fe corrosion products. FeCPs can be free or coated on sand. (Adapted from ref. ^70^).

System	Fe^0^	MnO_2_	Sand	Fe^0^/MnO_2_/Sand
t_0_ = 0	Fe^0^	MnO_2_	Sand	Fe^0^ + MnO_2_ + Sand
t > t_o_	Fe^0^ + FeCPs	MnO_2_ + MnOOH	Sand	Fe^0^ + MnO_2_ + Sand + FeCPs + MnOOH
t_∞_	FeCPs	MnOOH	Sand	MnOOH + Sand + FeCPs

In the Fe^0^/MnO_x_/sand system, iron and manganese chemically precipitate at the surface of MnO_x_, sand or in the bulk solution. Due to the good adsorptive affinities of Fe^2+^ and Fe^3+^ for sand surface, it is assumed that the deposition of Fe hydroxides at its surface (coating) will compete with Fe^2+^ consumption by the reductive dissolution of MnO_x_ until sand coating is completed. Thereafter, the “free” precipitation of iron and manganese occurs and the final products are deposits of more or less pure iron and manganese ores^66,71^. In other words, the investigated Fe^0^/MnO_x_/sand system is a ternary system only at the start of the experiment. It then turns to a mixture of Fe^0^, iron oxide-coated sand, iron oxide-coated MnO_x_, Fe/Mn shales, etc. Even after Fe^0^ depletion, the Fe/Mn mineral mixture will still be a reactive one, with a great potential for water treatment by both abiotic and biotic processes^67,68,72^.

### Experimental details

This experimental section is adapted from Cao et al.^70^ using the same experimental design and two more MnO_2_ minerals.

#### Solutions

The used methylene blue (MB—Basic Blue 9 from Merck) was of analytical grade. The working solution was 10.0 mg L^–1^ prepared by diluting a 1000 mg L^–1^ stock solution. The stock solution was prepared by dissolving accurately weighted MB in tap water. The use of tap water rather than deionised water was motivated by the fact that tap water is closer to natural water in its chemical composition. The MB molecular formula is C_16_H_18_N_3_SCl corresponding to a molecular weight of 319.85 g. MB was chosen in this study because of its well-known strong adsorption onto solids^70^.

#### Solid materials

##### Metallic iron (Fe^0^)

The used Fe^0^ material was purchased from iPutech (Rheinfelden, Germany). The material is available as filings with a particle size between 0.3 and 2.0 mm. Its elemental composition as specified by the supplier was: C: 3.52%; Si: 2.12%; Mn: 0.93%; Cr: 0.66% while the balance was Fe. The material was used without any further pre-treatment. Fe^0^ was proven as a powerful discoloration agent for MB given that discoloration agents in the form of FeCPs are progressively generated in-situ^70^.

##### Manganese dioxide (MnO_2_)

The tested natural MnO_2_-bearing minerals was Manganit from Ilfeld/Harz, Thüringen (Germany). The mineral was crushed and fractionated by sieving. The fraction 0.5–1.0 mm was used without any further pre-treatment. No chemical, mineralogical nor structural characterizations were performed. MnO_2_ is a reactive mineral^73–75^ and is used to delay the availability of ‘free’ iron corrosion products (FeCPs) in the system. This results in a delay of quantitative MB discoloration^30^.

##### Sand

The used sand was a commercial material for aviculture (“Papagaiensand” from RUT—Lehrte/Germany). The sand was used as received without any further pre-treatment. The particle size was between 2.0 and 4.0 mm. Sand was used as an adsorbent because of its worldwide availability and its use as admixing agent in Fe^0^ barriers^50,76^. The adsorption capacity of sand for MB has been systematically documented as early as in 1955 by Mitchell et al.^77^.

#### MB discoloration

Quiescent batch experiments (non-shaken) were conducted in assay tubes for experimental durations of 7, 18, 25 and 47 d. The batches consisted of 0.0 or 1.0 g of sand, 0.0 or 0.1 g to Fe^0^, 0.0 or 0.05 g of MnO_2_ and mixtures thereof in 22.0 mL of a 10.0 mg L^–1^ MB solution. The investigated systems were: (i) Fe^0^ alone, (ii) sand alone, (iii) MnO_2_ alone, (iv) Fe^0^/sand, (v) Fe^0^/MnO_2_ and (vi) Fe^0^/sand/MnO_2_. The efficiency of individual systems at discolouring MB was characterized at laboratory temperature (about 22 °C). Initial pH was about 8.2. After equilibration, up to 3.0 mL of the supernatant solutions were carefully retrieved (no filtration) for MB measurements (no dilution). Each experiment was performed in triplicates, and averaged values are presented. Table 3 summarizes the aggregate content of the 6 Fe^0^/MnO_2_/sand systems investigated herein. The operational reference (blank experiment) is also added. Note that the pure Fe^0^ system (Fe^0^ alone) is regarded as a ‘Fe^0^/MnO_2_/sand system’ without MnO_2_ nor sand.

**Table 3:** Overview on the six (6) investigated systems. The material loadings correspond to Fig. 2.

System	Fe^0^ (g L^−1^)	Sand (g L^−1^)	MnO_2_ (g L^−1^)	Materials	Comments
Reference	0.0	0.0	0.0	None	Blank experiment
System 1	4.5	0.0	0.0	Fe^0^ alone	Blank for Fe^0^
System 2	0.0	45.0	0.0	sand alone	Blank for sand
System 3	0.0	0.0	2.3	MnO_2_ alone	Blank for MnO_2_
System 4	4.5	45.0	0.0	Fe^0^/sand	Reference system
System 5	4.5	0.0	4.5	Fe^0^/MnO_2_	Reference system
System 6	4.5 to 45	45.0	4.5	Fe^0^/sand/MnO_2_	Fe^0^ loading as variable

**Figure 2:**
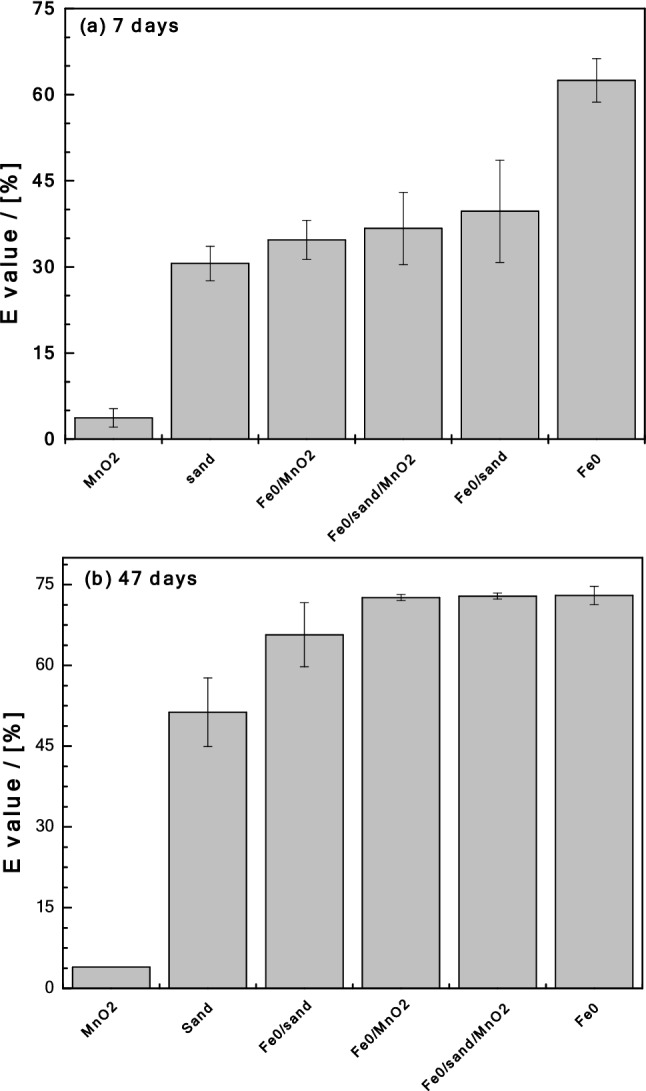
Comparison of the efficiency of tested materials for methylene blue (MB) discoloration for 7 (**a**) and 47 days (**b**). Experimental conditions: [Fe^0^] = 0 to 45 g L^–1^; [sand] = 45 g L^–1^; and [MnO_2_] = 2.3 g L^–1^.

#### Analytical methods

Iron and MB aqueous concentrations were determined by a Cary 50 UV–Vis spectrophotometer (Varian). The working wavelengths for MB and iron were 664.5 and 510.0 nm, respectively. Cuvettes with 1.0 cm light path were used. The spectrophotometer was calibrated for Fe and MB concentrations ≤ 10.0 mg L^–1^. The pH value was measured by combined glass electrodes (WTW Co., Germany).

#### Expression of MB discoloration results (E value)

In order to characterize the magnitude of the tested systems for MB discoloration, the discoloration efficiency (E) was calculated (Eq. 4). After the determination of the residual MB concentration (C), the corresponding percent MB discoloration (E value) was calculated as:

4 $${\text{E}} = \, [{1 } - \, ({\text{C}}/{\text{C}}_{0} )] \, \times { 1}00\% ,$$

where, C_0_ is the initial aqueous MB concentration (ideally 10.0 mg L^–1^), while C gives the MB concentration after the experiment. The operational initial concentration (C_0_) for each case was acquired from a triplicate control experiment without additive material (so-called blank). This procedure was to account for experimental errors during dilution of the stock solution, MB adsorption onto the walls of the reaction vessels, and all other possible side reactions during the experiments.

## Results and discussion

### Evidence for the complexity of the Fe^0^/MnO_2_/sand systems

Figure 2 compares the extent of MB discoloration in the six investigated systems for 7 and 47 days. Figure 2a clearly shows that, after 7 d, only MnO_2_ had not significantly discolored MB (4%) while Fe^0^ alone depicts the best discoloration efficiency (62%). The E values for the other systems varied between 31 and 40%. The increasing order of efficiency was: MnO_2_ < sand < Fe^0^/MnO_2_ < Fe^0^/MnO_2_/sand < Fe^0^/sand < Fe^0^. These results can be regarded as counter-intuitive since binary (Fe^0^/MnO_2_, Fe^0^/sand) and ternary (Fe^0^/MnO_2_/sand) performed less than Fe^0^ alone. In conventional shaken or stirred batch experiments, involved processes are accelerated to the extent that achieved results are the intuitive ones observed after 47 days (Fig. 2b)^78^.

Figure 2b compares the extent of MB discoloration in the six systems after 47 days. Compared to the results after 7 days, the extent of MB discoloration has increased to more than 50% in all systems, except MnO_2_ alone. Based on the absolute E values, the increasing order of efficiency was: MnO_2_ (4%) < sand (51%) < Fe^0^/sand (66%) < Fe^0^/MnO_2_ = Fe^0^/MnO_2_/sand = Fe^0^ (72%). It is interesting to note that Fe^0^/sand performed less than Fe^0^ alone and the two MnO_2_-bearing systems. This observation alone confirms that MnO_2_-amendment enhances the efficiency of Fe^0^/H_2_O systems by “reinforcing” corrosion (Eq. 3), but only in the long-term. Thus, the complexity of the ternary system as well as the need to understand its operation model is apparent. This is achieved herein by investigating the systems for 7, 18, 25 and 47 days. This corresponds to following the fate of aqueous MB (discoloration) as the contact time increases from 7 to 47 days^59,70,71^. In particular the variation of the pH value in the systems will be discussed in detail.

### Effect of the contact time on the Fe^0^/MnO_2_/sand system

**Figure 3:**
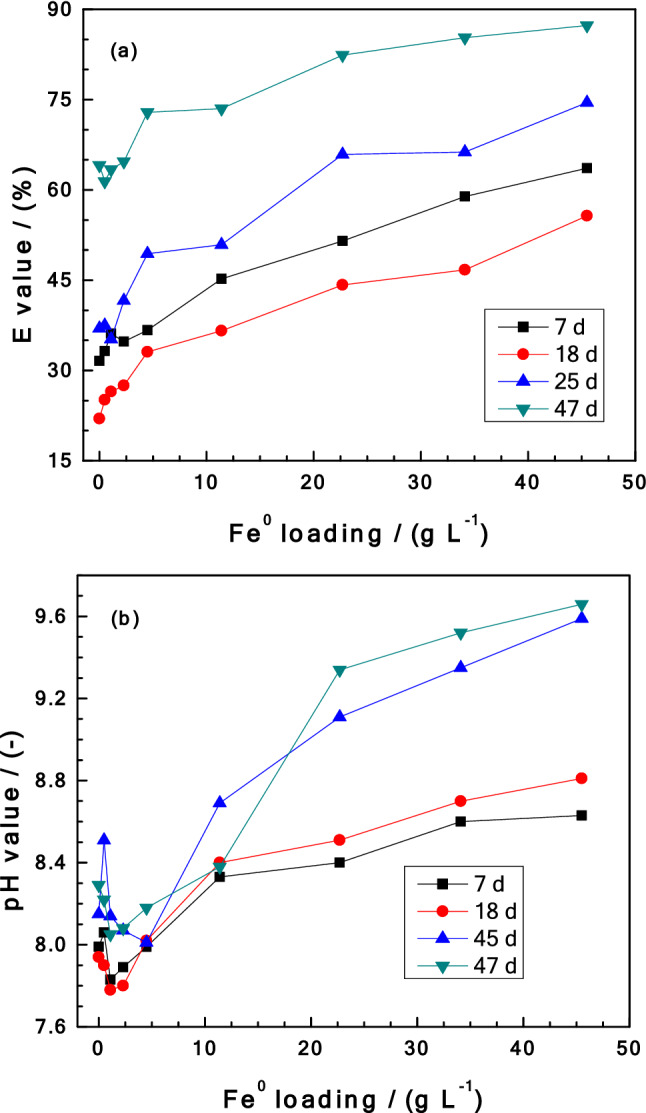
Changes in Fe^0^/sand/MnO_2_ systems as impacted by the addition of various Fe^0^ loading for 7, 18, 25 and 47 days: (**a**) Methylene blue discoloration, and (**b**) pH value. Experimental conditions: [Fe^0^] = 2.3 to 45 g L^–1^; [sand] = 22.5 g L^–1^; and [MnO_2_] = 2.3 g L^–1^. The lines are not fitting functions, they simply connect points to facilitate visualization.

Figure 3a compares the extent of MB discoloration in Fe^0^/MnO_2_/sand systems for the four tested contact times (7, 18, 25 and 47 d) and Fig. 3b depicts the corresponding changes in pH values. It is seen that the lowest extent of MB discoloration corresponds to 18 d contact time. This means that after 7 days the system performed better than after 18 d. The observation can be regarded as counter-intuitive, while the monotonous increase of the pH value (Fig. 3b) is intuitive. The investigated systems were 0 ≤ [Fe^0^] (g L^–1^) ≤ 45, with [MnO_2_] = 2.3 g L^–1^ and [sand] = 45 g L^–1^. This means that [Fe^0^] = 0.0 g L^–1^ corresponds to a MnO_2_/sand system or simplified to the sand system as MnO_2_ has no adsorptive affinities for MB (Fig. 2). In other words, the counter-intuitive observation corresponds to the effect of MnO_2_ on the Fe^0^/H_2_O system.

A key feature from Fig. 3a is that there is an intuitive monotonous increase of the E value with increasing Fe^0^ loading for all four tested contact times. This suggests that if the experiments were performed by different investigators, the given interpretations would have been conclusive and even convincing. The tested experimental conditions were selected based on past works^30,79^ to achieved such results. In addition, most of the observations made by researchers in Fe^0^/H_2_O systems are just static snap-shots (mostly inaccurately measured) of processes occurring over an enormous range of time scales^80^. Following this premise, it was necessary to further vary the experimental conditions to maximize the chance to make more relevant observation^81^. One really intriguing observation is that the MB previously removed (t < 7 days) was released back to the solution at day 18 such that MB discoloration was lower even in the sand system (Fig. 3a).

This MB desorption is rationalized by the pH decrease accompanying MnO_2_ reductive precipitation as given in Eq. (3b). By decreasing the pH value, further adsorption onto sand is inhibited and the previously adsorbed MB is desorbed and released into solution (Fig. 3a). On the other hand, the process of Fe^0^ dissolution by MnO_2_ implies intensified interactions at the bottom of the assay tubes which slowed down the diffusion of MB from the bulk solution. Iron corrosion determined the extent of MB discoloration and the intuitive increase of MB discoloration with increasing Fe^0^ loading is observed in all systems only after a pseudo-steady state is established in the systems or the capacity of MnO_2_ is exhausted. Alyoussef^59,70,71^ tested a parallel system with 4.5 g L^–1^ of MnO_2_ and observed a larger decrease of MB discoloration for 18 days. Similar observations were made by Noubactep et al.^82^ in their experiments for uranium removal.

Figure 3b shows that for [Fe^0^] > 7.5 g L^–1^, the pH value monotonously increases with increasing Fe^0^ loading. For [Fe^0^] < 7.5 g L^–1^, there were some fluctuations justified by the co-occurrence of Fe^0^ corrosion (consuming protons—Eq. 1) and MnO_2_ reductive dissolution (producing protons—Eq. 3b) to fix the pH of the systems. Again, once the oxidation capacity of MnO_2_ is exhausted, iron corrosion controls the pH of the system.

The comparative evaluation of the time-dependent changes of E and pH values has clarified the operating mode of MnO_2_ in enhancing the efficiency of the Fe^0^/H_2_O system without any solid phase characterization. This discussion has equally not considered the redox reactivity of MnO_2_ for Fe^0^ (and MB). Only the availability of “free” FeCPs was considered in the investigated single, binary and ternary aggregate systems. Achieved results corroborate the usefulness of varying several operational parameters to better understand complex dynamic systems^81,83,84^.

### Significance of the findings

#### Operating mode of remediation Fe^0^/H_2_O systems

This study has confirmed that Fe^0^ in engineered filtration systems is oxidized by protons to ferrous ion (Fe^2+^) (Eq. 1). Fe^2+^ ions are partly transferred to the surface of available aggregates (e.g. MnO_2_ and sand) and is oxidized further to ferric ion (Fe^3+^) and deposited on the aggregates as hydroxides (in-situ coating) (Fig. 1). Iron oxide-coated sand is a good adsorbent for several contaminants including chromium^39,85^, pathogens^86,87^ and phosphates^88,89^. Fe^0^ oxidation also contributes to produce anoxic conditions which are favorable for the abiotic reductive transformation of several dissolved species including chlorinated compounds^38,90^. Unlike sand and other inert aggregates, MnO_2_ is reactive and uses Fe^2+^ for its reductive dissolution (Eq. 3). Because the reaction occurs at the surface of MnO_2_ (Fig. 1), Fe^0^ passivation is delayed until the oxidative capacity of MnO_2_ is exhausted. Results presented herein have demonstrated these mechanisms excellently, while benefiting from the tracer nature of methylene blue (MB method)^30,79^. In fact, mechanistic discussions are often complicated by the need to consider the redox reactivity of both Fe^0^ and MnO_2_ with the contaminant of concern^72^. In other words, one major output of this research is that the popular hypothesis to rationalize reductive transformations in Fe^0^/H_2_O systems is faulty^91^. The hypothesis that Fe^0^ is an electron donor for dissolved has been seriously challenged during the past 15 years, however, the questioned view is still prevailing^11,36, 59^.

The stoichiometry of electrochemical reactions (similar to Eq. 2) has been routinely used to design Fe^0^ remediation systems^92,93^. The evidence that twice more Fe^0^ is needed to exchange the same number of electrons when reduction is induced by Fe^2+^ implies that the service life of Fe^0^-based systems has been wrongly estimated^57^. The statement is valid regardless of the approach used to estimate the efficiency of the system. However, the main problem has been the failure to properly consider the expansive nature of iron corrosion, which makes only hybrid systems viable in the long term^14^.

#### The importance of hybrid Fe^0^/H_2_O systems

The long history of Fe^0^ filtration systems teaches that only hybrid systems are sustainable. The Bischof filters, applied both for household and large-scale uses, contained a reactive zone made up of 25% sponge iron (vol/vol) mixed with gravel^41,94^. The Multi-Soil-Layering of Wakatsuki et al.^89^ contained only 15% Fe^0^ (w/w) (iron fillings) mixed with 15% Fe^0^ (w/w) pelletized jute and balanced with zeolite (60%). The phosphate filters of Erickson et al.^95^ contained only up to 5% steel wool balanced with sand. All these systems operated for more that 1 year without clogging. In the framework of subsurface permeable reactive barriers, O'Hannesin and Gillham^31^ tested a reactive wall containing 22% Fe^0^ balanced with gravel and reported on good hydraulic properties in the long term. Other systems with 100% Fe^0^ have failed because of loss of porosity coupled with the early development of preferential flow paths in the Fe^0^ permeable reactive barrier^96^. However, the availability of preferential flow paths was globally attributed to mineral precipitation (e.g. calcium carbonate, iron oxides, sulfide minerals). The key point is that iron oxides resulting from corrosion products are more abundant and universally present, and their generation should be reduced by “diluting” Fe^0^ with non-expansive aggregates like gravel or sand.

All systems containing a pure Fe^0^ layer (100%) were reported to be efficient but not sustainable^97–99^. The most prominent example is probably the use of iron filings for selenium removal from agricultural drainage water by the Harza Process^98,100^. In 1985, Harza Engineering Co. tested a pilot-scale process using iron filings in flow-through beds. The testing was discontinued because the beds quickly cemented with precipitates^100^. The study concluded that the advantage of Fe^0^ filtration is to decrease Se concentration to very low concentrations. The mechanism of Se removal was further investigated and it was established that Se is not reduced by an electrochemical mechanism^98^. Furthermore, Fe^0^ filters were suggested as a polishing step following microbial treatments^100^. Despite this evidence, it is disappointing to observe that available works on Se removal in Fe^0^/based systems have not built on existing knowledge as Se is still reported to be reduced by electrons from the metal body^60,101–103^. Following the state-of-the-art knowledge on the sustainability of Fe^0^ filtration, hybrid Fe^0^ systems should have been tested as stand-alone technology for Se removal. In essence, such work was independently conducted by Huang and his colleagues^15,43,104,105^ who developed and demonstrated the efficiency of a hybrid Fe^0^/Fe_3_O_4_ for the removal of several micro-pollutants, including Mo^VI^, NO_3_^–^ and Se^VI^, and recently for the mitigation of pathogens (bacteria) from dairy manure. The fact that a hybrid system, initially developed for chemically reducible micro-pollutants is performing well for pathogens corroborate the idea that it suffices to sustain iron corrosion to achieve water treatment^37,52–54,106^. As discussed in the “Introduction”, Huang et al.^43^ have not convincingly demonstrated the specificity of their hybrid system (Fe^0^/Fe_3_O_4_). This is particularly the case in a context where Fe^0^/sand systems are already essentially more sustainable than pure Fe^0^ (100%)^14,49^. The present work also confirms previous results that any additive to Fe^0^ basically delay the availability of corrosion products under typical field conditions. The observed enhanced performance results from sustained iron corrosion in the whole system. The question then arises, what makes MnO_2_ a specific admixing aggregate for Fe^0^ filters?

#### The suitability of hybrid Fe^0^/MnO_2_ systems

The presentation until now has demonstrated that applying Fe^0^ for water treatment is promising as mixing Fe^0^ with other aggregates delays passivation or sustain treatment efficiency. Moreover, substantial experiences have been accumulated on the functionality of hybrid systems for water treatment ("The importance of hybrid Fe^0^/H_2_O systems"). The knowledge that Fe^0^ acts as generator of contaminant scavengers (and never as reducing agent) implies that adsorption and co-precipitation are the fundamental mechanisms of contaminant removal in Fe^0^/H_2_O systems. Hybrid systems tested as means to prevent iron passivation include amendment with granular activated carbon (GAC), magnetite (Fe_3_O_4_), manganese oxides (MnO_x_), pyrite (FeS_2_), and sand^15^. Among these aggregates, MnO_x_ and FeS_2_ are the most chemically reactive^36,71^. Both aggregates induce a pH shift to more acidic values. However, because iron corrosion increases the pH, it is possible to find the optimal Fe^0^/FeS_2_ and/or Fe^0^/MnO_2_ ratio for case-specific water treatment. Therefore, long-term systematic testing with well-characterized materials is necessary.

Note that Fe^0^ is a generator of iron oxides, and adding Mn oxides (MnO_x_) to the system creates a very complex system, which is not new to geochemists, but which is yet to be investigated in the context of water treatment^68,72^. In fact, taken individually, the redox reactivity of these minerals plays important roles in the fate and transformation of many contaminants in natural environments^59,61–64,70–72^. Available works mostly investigate simple model systems with few contaminants^68,72^. To bridge the gap between simple model systems and complex environmental systems, a profound understanding of the redox reactivity of Mn- and Fe-oxides in complex model systems toward water decontamination is urgently needed. The effects of natural ligands (Cl^–^, HCO_3_^–^, PO_4_^3–^, SO_4_^2–^) and natural organic matter (NOM) on the redox reactivity of Fe^0^/MnO_2_ systems need to be investigated as well. Moreover, there is need to investigate the following: (i) fate of contaminants in Fe^0^ systems, and (ii) the safe disposal of spent Fe^0^ materials, including their use as filler material in novel construction materials, and the behavior of contaminants in such materials.

## Concluding remarks

This study clearly delineates the important role of reactive MnO_x_ minerals on the process of water treatment using Fe^0^-based systems. The presence of MnO_x_ induces Fe^2+^ oxidation at the mineral surface, resulting in a significant delay of Fe^0^ passivation compared to that attained in Fe^0^ and Fe^0^/sand systems. Being a natural mineral or a soil resource, its incorporation in Fe^0^ filters reinforces the frugality of this already demonstrated affordable system. It is expected that adding MnO_x_ to Fe^0^/H_2_O will create geochemical dynamics in the system which would sustain iron corrosion and maintain the efficiency of system for water decontamination for the long term. This would make Fe^0^ filters a sustainable solution for decentralized safe drinking water provision and enable the realization of universal access to safe drinking water and even on a self-reliant manner. To bridge the existing knowledge gaps, the need for further research entailing long-term testing of Fe^0^ systems was highlighted.

## References

[1] Schumacher EF (1973). Small is Beautiful: Economics as If People Mattered.

[2] Howe KJ, Hand DW, Crittenden JC, Trussell RR, Tchobanoglous G (2012). Principles of Water Treatment.

[3] Liu P, Gernjak W, Keller J (2017). Long-term performance of enhanced-zero valent iron for drinking water treatment: A lab-scale study. Chem. Eng. J..

[4] Tepong-Tsindé R, Ndé-Tchoupé AI, Noubactep C, Nassi A, Ruppert H (2019). Characterizing a newly designed steel-wool-based household filter for safe drinking water provision: Hydraulic conductivity and efficiency for pathogen removal. Processes.

[5] Nya, E. L. Access to drinking water and sanitation in Nde Division. Cameroon. PhD Dissertation, University of Yaoundé I. (2020).

[6] Nya EL, Mougoué B (2020). Access to safe drinking water and sanitary risks in the town of Bangangté (West Region of Cameroon). Saudi J. Hum. Soc. Sci..

[7] Banerji T, Chaudhari S, Nath K, Sharma V (2017). A cost-effective technology for arsenic removal: Case study of zerovalent iron-based IIT Bombay arsenic filter in West Bengal. Water and Sanitation in the New Millennium.

[8] Kearns JP, Bentley MJ, Mokashi P, Redmon JH, Levine K (2019). Underrepresented groups in WaSH—The overlooked role of chemical toxicants in water and health. J. Water Sanit. Hyg. Dev..

[9] Hussam A, Munir AKM (2007). A simple and effective arsenic filter based on composite iron matrix: Development and deployment studies for groundwater of Bangladesh. J. Environ. Sci. Health A.

[10] Antia DDJ, Kharissova OV (2020). Water treatment and desalination using the eco-materials n-Fe^0^ (ZVI), n-Fe_3_O_4_, n-Fe_x_O_y_H_z_[mH_2_O], and n-Fe_x_[Cation]_n_O_y_H_z_[Anion]_m_ [rH_2_O]. Handbook of Nanomaterials and Nanocomposites for Energy and Environmental Applications.

[11] Cao V, Yang H, Ndé-Tchoupé AI, Hu R, Gwenzi W, Noubactep C (2020). Tracing the scientific history of Fe^0^-based environmental remediation prior to the advent of permeable reactive barriers. Processes.

[12] Yang H (2020). Designing the next generation of Fe^0^-based filters for decentralized safe drinking water treatment. Processes.

[13] Huang Z, Cao V, Nya EL, Gwenzi W, Noubactep C (2021). Kanchan arsenic filters and the future of Fe^0^-based filtration systems for single household drinking water supply. Processes.

[14] Domga R, Togue-Kamga F, Noubactep C, Tchatchueng JB (2015). Discussing porosity loss of Fe^0^ packed water filters at ground level. Chem. Eng. J..

[15] Han S, Huang Y, Liu Z (2019). Bacterial indicator reduction in dairy manure using hybridzero-valent iron (h-ZVI) system. Environ. Sci. Pollut. Res..

[16] Henderson AD, Demond AH (2007). Long-term performance of zero-valent iron permeable reactive barriers: A critical review. Environ. Eng. Sci..

[17] Bartzas G, Komnitsas K (2010). Solid phase studies and geochemical modelling of low-cost permeable reactive barriers. J. Hazard Mater..

[18] Li L, Benson CH (2010). Evaluation of five strategies to limit the impact of fouling in permeable reactive barriers. J. Hazard Mater..

[19] Guan X (2015). The limitations of applying zero-valent iron technology in contaminants sequestration and the corresponding countermeasures: The development in zero-valent iron technology in the last two decades (1994–2014). Water Res..

[20] Gheju M, Balcu I (2021). Sequential abatement of Fe^II^ and Cr^VI^ water pollution by use of walnut shell-based adsorbents. Processes.

[21] Caré S (2013). Modeling the permeability loss of metallic iron water filtration systems. Clean: Soil, Air, Water.

[22] Moraci N, Lelo D, Bilardi S, Calabrò PS (2016). Modelling long-term hydraulic conductivity behaviour of zero valent iron column tests for permeable reactive barrier design. Can. Geotech. J..

[23] Li J (2019). Characterization methods of zerovalent iron for water treatment and remediation. Water Res..

[24] Lufingo M, Ndé-Tchoupé AI, Hu R, Njau KN, Noubactep C (2019). A novel and facile method to characterize the suitability of metallic iron for water treatment. Water.

[25] Romanoff, M. Underground Corrosion. United States Department of Commerce, National Bureau of Standards. Circular 579 (1957).

[26] Melchers RE, Petersen RB (2018). A reinterpretation of the Romanoff NBS data for corrosion of steels in soils. Corros. Eng. Sci. Technol..

[27] Stefanonia M, Zhanga Z, Angsta U, Elsener B (2018). The kinetic competition between transport and oxidation of ferrous ions governs precipitation of corrosion products in carbonated concrete. RILEM Tech. Lett..

[28] Stefanoni M, Angst UM, Elsener B (2019). Kinetics of electrochemical dissolution of metals in porous media. Nat. Mater..

[29] Whitney WR (1903). The corrosion of iron. J. Am. Chem. Soc..

[30] Miyajima K, Noubactep C (2015). Characterizing the impact of sand addition on the efficiency of granular iron for water treatment. Chem. Eng. J..

[31] O'Hannesin SF, Gillham RW (1998). Long-term performance of an in situ "iron wall" for remediation of VOCs. Ground Water.

[32] Phillips DH (2010). Ten year performance evaluation of a field-scale zero-valent iron permeable reactive barrier installed to remediate trichloroethene contaminated groundwater. Environ. Sci. Technol..

[33] Wilkin RT (2014). Fifteen-year assessment of a permeable reactive barrier for treatment of chromate and trichloroethylene in groundwater. Sci. Tot. Environ..

[34] Wilkin RT (2019). Geochemical and isotope study of trichloroethene degradation in a zero-valent iron permeable reactive barrier: A twenty-two-year performance evaluation. Environ. Sci. Technol..

[35] Roh Y, Lee SY, Elless MP (2000). Characterization of corrosion products in the permeable reactive barriers. Environ. Geol..

[36] Hu R, Cui X, Xiao M, Gwenzi W, Noubactep C (2021). Characterizing the impact of pyrite addition on the efficiency of Fe^0^/H_2_O systems. Sci. Rep..

[37] Noubactep C (2007). Processes of contaminant removal in “Fe^0^–H_2_O” systems revisited. The importance of co-precipitation. Open Environ. Sci..

[38] Jiao Y (2009). Reductive dechlorination of carbon tetrachloride by zero-valent iron and related iron corrosion. Appl. Catal. B Environ..

[39] Gheju M (2011). Hexavalent chromium reduction with zero-valent iron (ZVI) in aquatic systems. Water Air Soil Pollut..

[40] Ghauch A (2015). Iron-based metallic systems: An excellent choice for sustainable water treatment. Freiberg Online Geosci..

[41] Bischof G (1973). On the purification of water. Proc. R. Philos. Soc. Glasgow.

[42] Mackenzie PD, Horney DP, Sivavec TM (1999). Mineral precipitation and porosity losses in granular iron columns. J. Hazard Mater..

[43] Huang YH, Tang C, Zeng H (2012). Removing molybdate from water using a hybridized zero-valent iron/magnetite/Fe(II) treatment system. Chem. Eng. J..

[44] Noubactep C, Meinrath G, Dietrich P, Merkel B (2003). Mitigating uranium in groundwater: Prospects and limitations. Environ. Sci. Technol..

[45] Noubactep C, Btatkeu-K BD, Tchatchueng JB (2011). Impact of MnO_2_ on the efficiency of metallic iron for the removal of dissolved metal. Chem. Eng. J..

[46] Lipczynska-Kochany E, Harms S, Milburn R, Sprah G, Nadarajah N (1994). Degradation of carbon tetrachloride in the presence of iron and sulphur containing compounds. Chemosphere.

[47] Westerhoff P, James J (2003). Nitrate removal in zero-valent iron packed columns. Water Res..

[48] Bi E, Devlin JF, Huang B (2009). Effects of mixing granular iron with sand on the kinetics of trichloroethylene reduction. Ground Water Monit. Remed..

[49] Song DI, Kim YH, Shin WS (2005). A simple mathematical analysis on the effect of sand in Cr(VI) reduction using zero valent iron. Korean J. Chem. Eng..

[50] Ndé-Tchoupé AI, Makota S, Nassi A, Hu R, Noubactep C (2018). The suitability of pozzolan as admixing aggregate for Fe^0^-based filters. Water.

[51] Bigg T, Judd SJ (2000). Zero-valent iron for water treatment. Environ. Technol..

[52] Furukawa Y, Kim J-W, Watkins J, Wilkin RT (2002). Formation of ferrihydrite and associated iron corrosion products in permeable reactive barriers of zero-valent iron. Environ. Sci. Technol..

[53] Purenović M, Perović J, Bojić A, Andelković T, Bojić D (2004). Cu and Cd removal from wastewater by a microalloyed aluminium composite. Environ. Chem. Lett..

[54] Burghardt D, Kassahun A (2005). Development of a reactive zone technology for simultaneous in situ immobilisation of radium and uranium. Environ. Geol..

[55] Yoon IH, Kim KW, Bang S, Kim MG (2011). Reduction and adsorption mechanisms of selenate by zero-valent iron and related iron corrosion. Appl. Catal. B Environ..

[56] Xu J (2019). Reactivity, selectivity, and long-term performance of sulfidized nanoscale zerovalent iron with different properties. Environ. Sci. Technol..

[57] Cao V, Ndé-Tchoupé AI, Hu R, Gwenzi W, Noubactep C (2021). Discussing the mechanism of contaminant removal in Fe^0^/H_2_O systems: The burden of a poor literature review. Chemosphere.

[58] Zhang L, Zhang Y, Gao X, Xu C (2019). Insights on the effects of pH and Fe(II) regeneration during the chromate sequestration by sulfidated zero-valent iron. Chem. Eng. J..

[59] Alyoussef G (2021). Characterizing the impact of contact time in investigating processes in Fe^0^/H_2_O systems. Freiberg Online Geosci..

[60] Wang X, Zhang X, Wang Z, Xu C, Tratnyek PG (2021). Advances in metal(loid) oxyanion removal by zerovalent iron: Kinetics, pathways, and mechanisms. Chemosphere.

[61] Appelo CAJ, Postma D (1999). Variable dispersivity in a column experiment containing MnO_2_ and FeOOH-coated sand. J. Cont. Hydrol..

[62] Appelo CAJ, Postma D (1999). A consistent model for surface complexation on birnessite (MnO_2_) and its application to a column experiment. Geochim. Cosmochim. Acta.

[63] Post JE (1999). Manganese oxide minerals: Crystal structures and economic and environmental significance. Proc. Natl. Acad. Sci..

[64] Tebo BM (2004). Biogenic manganese oxides: Properties and mechanisms of formation. Annu. Rev. Earth Planet Sci..

[65] Vodyanitskii YN (2009). Mineralogy and geochemistry of manganese: A review of publications. Eurasian Soil Sci..

[66] Penrose RAF (1893). The chemical relation of iron and manganese in sedimentary rocks. J. Geol..

[67] Fischel MHH, Fischel JS, Lafferty BJ, Sparks DL (2015). The influence of environmental conditions on kinetics of arsenite oxidation by manganese-oxides. Geochem. Trans..

[68] Huang J, Zhang H (2020). Redox reactions of iron and manganese oxides in complex systems. Front. Environ. Sci. Eng..

[69] Lewis AE (2010). Review of metal sulphide precipitation. Hydrometallurgy.

[70] Cao V, Alyoussef G, Gatcha-Bandjun N, Gwenzi W, Noubactep C (2021). The suitability of methylene blue discoloration (MB method) to investigate the Fe^0^/MnO_2_ system. Processes.

[71] Cao V, Alyoussef G, Gatcha-Bandjun N, Gwenzi W, Noubactep C (2021). Characterizing the impact of MnO_2_ addition on the efficiency of Fe^0^/H_2_O systems. Sci. Rep..

[72] Michel MM (2020). Mineral materials coated with and consisting of MnO_x_—Characteristics and application of filter media for groundwater treatment: A review. Materials.

[73] Shindo H, Huang PM (1984). Catalytic effects of manganese(IV), iron(III), aluminum, and silicon oxides on the formation of phenolic polymers. Soil Sci. Soc. Am. J..

[74] Sparrow LA, Uren NC (2014). Manganese oxidation and reduction in soils: Effects of temperature, water potential, pH and their interactions. Soil Res..

[75] Ye Z, Giraudon JM, De Geyter N, Morent R, Lamonier JF (2018). The design of MnO*x* based catalyst in post-plasma catalysis configuration for toluene abatement. Catalysts.

[76] Varlikli C (2009). Adsorption of dyes on Sahara desert sand. J. Hazard Mater..

[77] Mitchell G, Poole P, Segrove HD (1955). Adsorption of methylene blue by high-silica sands. Nature.

[78] Kurth, A. M. Discoloration of methylene blue by elemental iron—Influence of the shaking intensity. Bachelor thesis, Universität Göttingen, 45 (2008).

[79] Miyajima K (2012). Optimizing the design of metallic iron filters for water treatment. Freiberg Online Geosci..

[80] Brenner S (2010). Sequences and consequences. Philos. Trans. R. Soc. B.

[81] Lavine BK, Auslander G, Ritter J (2001). Polarographic studies of zero valent iron as a reductant for remediation of nitroaromatics in the environment. Microchem. J..

[82] Noubactep C, Schöner A, Meinrath G (2006). Mechanism of uranium (VI) fixation by elemental iron. J. Hazard Mater..

[83] Gatcha-Bandjun N, Noubactep C, Loura-Mbenguela B (2017). Mitigation of contamination in effluents by metallic iron: The role of iron corrosion products. Environ. Technol. Innov..

[84] Touomo-Wouafo M (2020). Electrochemical monitoring of heavy metals removal from aqueous solutions by aged metallic iron. Competitive effects of cations Zn^2+^, Pb^2+^ and Cd^2+^. Monatsh. Chem..

[85] Powell MR, Puls WR, Hightower KS, Sebatini AD (1995). coupled iron corrosion and chromate reduction: Mechanisms for subsurface remediation. Environ. Sci. Technol..

[86] Bojic A (2001). The inactivation of *Escherichia coli* by microalloyed aluminium based composite. Facta Universitatis.

[87] You Y, Han J, Chiu PC, Jin Y (2005). Removal and inactivation of waterborne viruses using zerovalent iron. Environ. Sci. Technol..

[88] James BR, Rabenhorst MC, Frigon GA (1992). Phosphorus sorption by peat and sand amended with iron oxides or steel wool. Water Environ. Res..

[89] Wakatsuki T, Esumi H, Omura S (1993). High performance and N, P removable on-site domestic wastewater treatment system by multi-soil-layering method. Water Sci. Technol..

[90] Khudenko BM (1991). Feasibility evaluation of a novel method for destruction of organics. Water Sci. Technol..

[91] Matheson LJ, Tratnyek PG (1994). Reductive dehalogenation of chlorinated methanes by iron metal. Environ. Sci. Technol..

[92] Sarr D (2001). Zero-valent-iron permeable reactive barriers—How long will they last?. Remediation.

[93] Obiri-Nyarko F, Grajales-Mesa SJ, Malina G (2014). An overview of permeable reactive barriers for in situ sustainable groundwater remediation. Chemosphere.

[94] Devonshire E (1890). The purification of water by means of metallic iron. J. Frankl. Inst..

[95] Erickson AJ, Gulliver JS, Weiss PT (2007). Enhanced sand filtration for storm water phosphorus removal. J. Environ. Eng..

[96] Morrison SJ, Mushovic PS, Niesen PL (2006). Early breakthrough of molybdenum and uranium in a permeable reactive barrier. Environ. Sci. Technol..

[97] Murphy AP (1988). Removal of selenate from water by chemical reduction. Ind. Eng. Chem. Res..

[98] Anderson, M. A. Fundamental aspects of selenium removal by Harza process. Rep San Joaquin Valley Drainage Program, US Dep Interior, Sacramento (1989).

[99] Hussam A (2009). Contending with a development disaster: Sono filters remove arsenic from well water in Bangladesh. Innovations.

[100] Frankenberger WT (2004). Advanced treatment technologies in the remediation of seleniferous drainage waters and sediments. Irrig. Drain. Syst..

[101] Liang L (2015). Efficient selenate removal by zero-valent iron in the presence of weak magnetic field. Sep. Purif. Technol..

[102] Liang L (2015). Kinetics of selenite reduction by zero-valent iron. Desalin. Water Treat..

[103] Qin H (2018). Unexpected effect of buffer solution on removal of selenite and selenate by zerovalent iron. Chem. Eng. J..

[104] Huang YH, Peddi PK, Zeng H, Tang C-L, Teng X (2013). Pilot-scale demonstration of the hybrid zero-valent iron process for treating flue-gas-desulfurization wastewater: Part I. Water Sci. Technol..

[105] Huang YH, Peddi PK, Zeng H, Tang C-L, Teng X (2013). Pilot-scale demonstration of the hybrid zero-valent iron process for treating flue-gas-desulfurization wastewater: Part II. Water Sci. Technol..

[106] Noubactep C (2008). A critical review on the mechanism of contaminant removal in Fe^0^–H_2_O systems. Environ. Technol..

